# How Aesthetic and Economic Factors Affect Consumers’ Purchasing Decisions: Evidence from EEG

**DOI:** 10.3390/bs16101819

**Published:** 2026-10-04

**Authors:** Shaohua Kong, Hanzun Li, Hongyu Fang

**Affiliations:** 1 Cultural and Economic Research Institute, Central University of Finance and Economics, Beijing 100081, China; tiger_ksh@163.com; 2 Business School, China University of Political Science and Law, Beijing 100088, China; 3 School of Economics and Management, Xizang University, Lhasa 850000, China

**Keywords:** artwork, aesthetic perception, price, purchasing decision, electroencephalogram (EEG)

## Abstract

Consumer theory identifies cultural and economic factors as key influences on purchasing decisions. Artworks, as material carriers of culture, are shaped by these factors in a particularly pronounced way. This study proposes that consumers’ artwork purchasing decisions are jointly determined by aesthetic and economic considerations. Using event-related potential (ERP) technology, artworks were classified into a 2 by 2 matrix of aesthetic valence and price. Seventy students from a university in western China made purchase or rejection decisions while their electroencephalogram was recorded. Results showed that both aesthetic and price factors jointly influenced artwork purchasing decisions. Aesthetic valence had a larger effect (*F*(1, 69) = 134.98, *p* < 0.001, *ηp*^2^ = 0.662) than price (*F*(1, 69) = 22.47, *p* < 0.001, *ηp*^2^ = 0.246), and participants were most willing to buy positively rated, low-priced artworks (*M* = 42.2%, *SD* = 24.2). The medial frontal negativity (MFN) amplitude and theta-band power were larger during purchase than rejection decisions, and this difference was most pronounced when participants chose to buy negatively rated artworks (*t*(48) = 4.31, *p* < 0.001, *d* = 0.61). Because the sample was drawn from a single university in western China and consisted of young students, the conclusions carry generalisability limitations. This study maps aesthetic and economic considerations during consumer purchasing onto separable neural processes, contributing to the intersection of cultural neuroscience and neuroeconomics.

## 1. Introduction

Maslow’s hierarchy of needs proposes that as economies and societies develop, people move beyond basic material necessities and pay greater attention to spiritual and cultural needs such as esteem and self-actualisation (Wahba & Bridwell, 1976). Art is one of the principal avenues through which people acquire social aesthetic and moral values (Rusu, 2017), and it fosters interaction between the viewer and the artwork, thereby supporting personal psychological, spiritual, and emotional fulfilment (Faubion & Rabinow, 1995). Consumer theory notes that in art consumption, factors such as aesthetic and monetary value, personal taste, and social reputation all influence willingness to purchase art (Spee et al., 2022). Among these, aesthetic perception plays a central role. Aesthetic perception refers to the understanding of an artwork’s aesthetic features, encompassing observable internal characteristics such as imitation, form, and expressiveness, as well as external characteristics such as historical, social, and institutional context (Haanstra, 1996). On this basis, people form aesthetic experiences and aesthetic judgments, mobilising emotion, imagination, and association while anchoring the evaluation in their own cultural background and arriving at a rational or affective confirmation of the aesthetic object (Watson et al., 1993). This understanding of aesthetic features provides consumers with an initial grasp of art and forms the basis for artwork purchasing decisions.

As a cultural commodity, the economic value of art is another important factor shaping consumption. This value is typically expressed through the price of the artwork. Price has long been regarded as a key determinant of consumer decisions; it is the monetary cost consumers incur to satisfy a consumption need (Hoseason, 2003). Price therefore represents an economic burden, and higher prices tend to depress purchase intention. On the other hand, price also serves as a signal of product quality, with higher prices implying better quality (Zeithaml, 1988). Price further reflects the symbolic differences carried by art, and these symbols satisfy consumers’ pursuit of status and reputation (Thornton, 2008). In art consumption, price is the market-based monetary expression of value and reflects the circulation characteristics of the art market.

In sum, when people decide whether to buy an artwork, two considerations operate simultaneously: whether they like the piece (aesthetic attitude) and whether the price is worth paying (economic attitude). Existing neuroaesthetics research has focused primarily on aesthetic attitude, while existing neuroeconomics research has focused primarily on economic attitude. Few studies have placed the two within a single framework and observed their interaction through the act of purchase, even though both genuinely shape consumer decisions in actual art transactions. This study draws on the consumer-theory view that purchase behaviour is driven by attitudes toward the product itself and toward the transaction and proposes that artwork purchasing decisions are influenced by both aesthetic and economic attitudes. ERP technology is used to observe how these two attitudes jointly shape the decision process.

## 2. Literature Review and Hypotheses

Artworks carry rich cultural value (Colbert & St-James, 2014), social value, worldview value (Faubion & Rabinow, 1995), and emotional value. For artworks that also function as commodities, providing consumers with aesthetic added value is an important way to stand out from competitors (Mata et al., 2013). People encounter art through aesthetic perception, a response that combines cognitive, affective, behavioural, and physiological components (Tschacher et al., 2012). Building on Solso’s theories of prior cognition and directed cognition and on Latto’s notion of aesthetic primitives, Zeki treated aesthetics as the earliest visual response to objects and proposed that it shapes subsequent aesthetic experience and judgement (Latto, 1995; Martindale & Moore, 1988; Solso, 1994; Zeki, 1993). With respect to aesthetic experience, Chatterjee and Vartanian (2016) proposed a model of conscious pleasure in aesthetics, and F. Zhou (2023) discussed four types of aesthetic experience on this basis, examining embodied simulation during aesthetic appreciation. Zhang and Zhou (2021) characterised aesthetic experience as immediate, dynamic, individual, and empathic. With respect to aesthetic judgement, Ishizu and Zeki (2013) identified neural links among aesthetic experience, aesthetic judgement, and cognitive judgement. M. Zhou and Feng (2014) further showed that aesthetic judgement comprises affective, reward-related, contextual, and knowledge-based mechanisms. Subsequent neuroaesthetics research has addressed literary aesthetics (Bara et al., 2021), aesthetic pleasure processing (Zhang & Zhou, 2021), aesthetic judgement of dynamic landscapes (Era et al., 2015), mate preference (Chatterjee & Vartanian, 2016), negative affect and the sublime (Ha & Lennon, 2010), and reward-related sensorimotor processing (Kirsch et al., 2016), but few studies have integrated neuroaesthetics with neuroeconomics.

As a material carrier of culture, art is also a prominent commodity in contemporary society (Li, 2024). Mata et al. (2013) reported a significant association between the desire to own a product and its perceived qualities. In consumption, aesthetic perception is an integrated activity of body and mind (Fetz, 2012). When viewing an artwork, the observer first grasps it visually, forms an impression through embodiment and mental cognition, and then produces experiential and behavioural responses (Iacoboni & Dapretto, 2006; Molnar-Szakacs & Uddin, 2013; M. Zhou & Feng, 2014). This aesthetic response is rapid and unconscious and can carry positive or negative bias (Iacoboni & Dapretto, 2006). Aesthetic perception therefore shapes purchasing decisions in actual art consumption, and because individuals differ in their aesthetic perception, this factor was difficult to quantify before the emergence of neuroscience methods.

Other factors influencing art consumption can be grouped into individual and social factors; their joint influence shapes purchasing decisions (Sun et al., 2020), and price is regarded as one of the most important elements. Individual factors relate mainly to knowledge of or familiarity with artworks (Mastandrea et al., 2009). Chatterjee and Vartanian (2016) proposed that aesthetic experience arises from interactions among affective, sensorimotor, and meaning systems, although this mechanism has not been fully supported by data. Holbrook (1999) argued that buyers rely more on their own pleasure and satisfaction than on objective product benefits. Social factors include cultural environment (Ha & Lennon, 2010), conformity pressure (Hesslinger et al., 2017), art criticism (Light & Odden, 2017), a sense of responsibility and mission (Li, 2024), emotion (Mowen & Carlson, 2003), social exclusion (Berns et al., 2010), and price (Beckert & Rössel, 2013; Eaton, 1995; Riemer et al., 2014). Rao and Monroe (1988) found that in multi-cue settings, price is often the dominant factor in consumer purchasing decisions. In economic theory, price expresses willingness to pay for a specific good. Neoclassical microeconomics holds that price conveys quality information and is one expression of product quality (Stiglitz, 1987). De Silva et al. (2012) argued that artworks differ from ordinary commodities in their future resale value and the emotional utility of ownership, and that artistic value is positively associated with price. Grampp noted that art is an economic good whose value emerges from the market; typically, higher prices signal better perceived quality and stronger purchase intention, but when price exceeds the buyer’s budget, the purchase is abandoned.

In sum, prior work has established that aesthetic factors, represented by aesthetic perception, and economic factors, represented by price, both influence artwork purchasing decisions. However, methodological limitations have made it difficult to measure and compare aesthetic factors directly. The emergence of neuroaesthetics addressed this limitation by developing methods such as fMRI (Zhao et al., 2020), functional magnetic resonance imaging (Ishizu & Zeki, 2011), anterior cingulate cortex recording (Bara et al., 2021), and event-related potentials, enabling quantitative study of brain activity. Existing neuroaesthetics research has rarely engaged neuroeconomics, leaving unresolved questions about how aesthetic and economic attitudes jointly influence purchasing decisions, which exerts a stronger effect, and how they interact.

The present study uses a 2 by 2 framework to examine how aesthetic and economic attitudes jointly shape artwork purchasing decisions. Aesthetically, artworks are divided into beautiful and ugly; economically, prices are divided into high and low. Participants view an artwork and its price, decide whether to buy it, and their EEG is recorded. Behaviourally, this yields the independent and joint effects of aesthetic and economic attitudes on purchase rates. Neurally, it yields differences in brain activity associated with the two attitudes during the decision process.

Based on prior research and the present design, five predictions were formulated.

**Hypothesis 1** **(H1).** *Price influences purchase intention, such that low-priced artworks are purchased more often than high-priced ones.*

**Hypothesis 2** **(H2).** *Aesthetic value influences purchase intention, such that positively rated artworks are purchased more often than negatively rated ones.*

**Hypothesis 3** **(H3).** *Aesthetic and economic attitudes jointly influence purchasing decisions, such that an interaction effect exists.*

**Hypothesis 4** **(H4).** *MFN amplitude differs between purchase and rejection decisions, and this difference is modulated by the aesthetic and economic attributes of the artwork.*

**Hypothesis 5** **(H5).** *Theta-band power differs between purchase and rejection decisions, and this difference is modulated by the aesthetic and economic attributes of the artwork.*

## 3. Materials and Methods

### 3.1. Participants

Seventy students (35 women, 35 men; age *M* = 22.6, *SD* = 2.6, range 19–31 years) from a university in western China participated in the experiment in exchange for payment. All were right-handed, had normal or corrected-to-normal vision, and reported no history of neurological or psychiatric disorder. The study was approved by the Ethics Review Committee of the Central University of Finance and Economics (IRB20250427002, 27 April 2025) and was conducted in accordance with the Declaration of Helsinki. Data collection took place at a partner university, and all participants provided written informed consent.

### 3.2. Stimuli

Four hundred and eighty artwork images were selected from the National Art Museum of China, Arts&Culture, and the China Art Exhibition Hall, and were uniformly edited for size and resolution.

To classify artworks as beautiful or ugly, nine raters were recruited from the same western Chinese university. Raters were not art majors and had received no specialised art training. There were four men and five women, aged 20 to 28, with normal or corrected-to-normal vision. Raters did not take part in the EEG experiment. Images were presented in a group testing session for 5 s each, and each rater independently judged whether the image was beautiful or ugly. For each image, the number of raters judging it as beautiful was counted. The 480 images were then ranked; the 96 images rated beautiful by the most raters (top 20%) formed the beautiful set, and the 96 images rated beautiful by the fewest raters (bottom 20%) formed the ugly set, yielding 192 experimental images. Inter-rater reliability for the beautiful/ugly judgments was ICC(2,9) = 0.84 for the mean rating and ICC(2,1) = 0.36 for a single rater, indicating good reliability at the group level but only moderate agreement for individual raters. Fleiss’ kappa for the nine-way classification was 0.41, representing moderate agreement beyond chance.

Whether an artwork felt expensive or cheap depended on both the absolute price and the viewer’s income. This study therefore used the ratio of artwork price to annual household income as the relative price measure. Artwork prices were based on actual market sale or auction values averaged across sources. The price ranges differed substantially across sources. Artworks from the National Art Museum of China and the China Art Exhibition Hall had a mean market value equal to 86 times annual household income and were defined as high-priced. Artworks from Arts&Culture had a mean market value equal to one-tenth of annual household income and were defined as low-priced.

At the individual level, because different individuals have different income and spending levels, their subjective sense of a given price also differs. For individuals with higher income and consumption, an absolute price that is objectively high may feel relatively low. Income and spending levels were therefore used as proxy variables for relative price when examining their associations with neural measures.

### 3.3. Procedure

**Figure 1:**
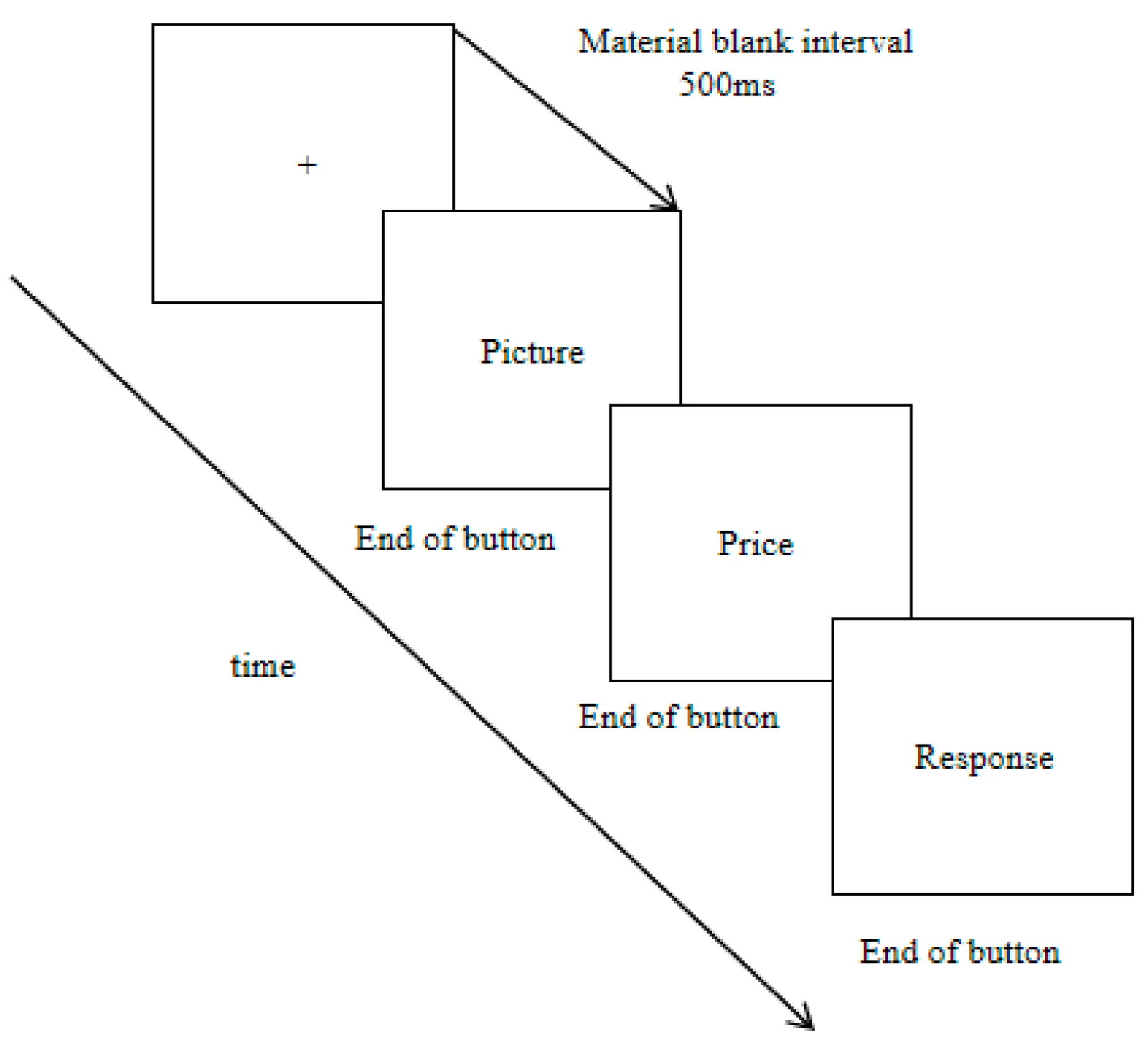
Schematic of the experimental paradigm.

The experimental paradigm is illustrated in Figure 1. Each trial began with a fixation cross presented for 1000 ms. Participants first made an aesthetic judgement (beautiful or ugly) of a randomly presented artwork image. After this judgement, they entered their annual household income and pressed the space bar. Following a 500 ms blank interval, a randomly selected image that the participant had judged as beautiful or ugly appeared on screen, with the price displayed below. Participants then decided whether to purchase the artwork: pressing 1 to buy and 2 to reject. Responses were required within 4000 ms, after which the trial advanced automatically. The inter-trial interval was 500 ms. There were 120 trials in total, 30 per condition, with no practice trials. The four conditions were presented in random order such that trials of the same condition never appeared consecutively. On average, each trial lasted approximately 4.5 s, the task lasted approximately 10 min per participant, and EEG electrode preparation took approximately 50 min per participant. In total, the experiment took approximately 70 h, and data collection was completed over a four-month period.

### 3.4. EEG Recording

EEG was recorded from a 64-channel Ag/AgCl electrode cap arranged according to the extended international 10–20 system, using a NeuroScan amplifier. Online recording was referenced to the right mastoid. Signals were sampled at 500 Hz and online bandpass filtered from 0.05 to 100 Hz. All electrode impedances were kept below 5 kΩ. Horizontal electrooculography (HEOG) was recorded from the outer canthi of both eyes, and vertical electrooculography (VEOG) from above and below the left eye.

### 3.5. EEG Analysis

#### 3.5.1. Preprocessing

EEG data were preprocessed using MATLAB R2023b (The MathWorks, Natick, MA, USA) and EEGLAB 2024.2 (Swartz Center for Computational Neuroscience, La Jolla, CA, USA). EEG data from 21 channels (Fp1, Fpz, Fp2, F7, F3, Fz, F4, F8, T7, C3, Cz, C4, T8, P7, P3, Pz, P4, P8, O1, Oz, O2) were extracted for analysis. Offline data were re-referenced using the reference electrode standardisation technique (REST). Independent component analysis (ICA) was used to remove ocular artifacts. A 0.1 to 30 Hz offline bandpass filter was applied, a range that covers the frequencies typically retained for medial frontal components. The analysis epoch spanned from 200 ms before to 1000 ms after stimulus onset, with the 200 ms pre-stimulus interval serving as the baseline. Trials with amplitudes exceeding ±100 μV were rejected.

#### 3.5.2. ERP Components

After preprocessing, artifact-free trials were averaged separately for each participant. Before conducting hypothesis tests, difference waves (purchase minus rejection) were computed at all 21 analysis channels. At each channel, multiple sliding windows between 200 and 400 ms post-stimulus were tested in 50 ms steps, and the window and channel with the largest decision effect were recorded. Although MFN and feedback-related negativity (FRN) components are typically maximal at midline frontocentral sites, in the present data, the purchase–rejection difference wave peaked at the right frontal electrode F4, where the 220 to 350 ms window showed the clearest separation. Component amplitude was therefore defined as the mean voltage at F4 within this window.

#### 3.5.3. Power Spectral Density

Power spectral density (PSD) describes how signal power is distributed across frequency bands. PSD was computed using the Welch method implemented in MATLAB (R2023b; The MathWorks, Natick, MA, USA). The Welch algorithm was used to compute PSD for each participant and channel. The signal was divided into segments, the squared magnitude of the discrete Fourier transform was computed for each segment using a window function, and the segment periodograms were averaged. A Hamming window was applied, with 50% overlap between consecutive 500 ms windows. When expected responses need adjustment and conflict arises between product expectations and price information, theta-band power increases (Cavanagh & Frank, 2014; Horr et al., 2022; Yang et al., 2022). The present study therefore focused on the 4 to 7 Hz theta band and did not examine delta, alpha, beta, or gamma bands.

#### 3.5.4. Statistical Analysis

Purchase rates were analysed using a 2 (price: high vs. low) by 2 (aesthetic valence: beautiful vs. ugly) repeated-measures ANOVA, reporting main effects, the interaction, and partial eta-squared (*ηp*^2^). Statistical analyses were performed using **SPSS 26.0 (IBM Corp., Armonk, NY, USA). Pairwise comparisons among the four conditions used paired-sample *t*-tests, with Cohen’s d as the effect size. EEG data were grouped by artwork type and decision: buy beautiful (BY), reject beautiful (BN), buy ugly (UY), and reject ugly (UN). MFN amplitude and theta power were compared across these groups using paired-sample *t*-tests. Because some participants had no ugly-art purchase trials, the UY analysis included 49 participants and the BY analysis included 67. All behavioural pairwise comparisons, EEG paired contrasts, and channel-wise difference maps were corrected using the Benjamini–Hochberg false discovery rate (FDR) procedure. Pearson correlations were computed to examine associations among neural and behavioural measures and individual price-reference variables; these correlations were exploratory and conducted post hoc, so uncorrected exact *p*-values are reported. Table 1 reports the number of artifact-free trials per condition, and Table 2 reports the sample size used in each analysis. Post hoc power analyses were conducted using G*Power 3.1.

## 4. Results

### 4.1. Behavioural Results

**Figure 2:**
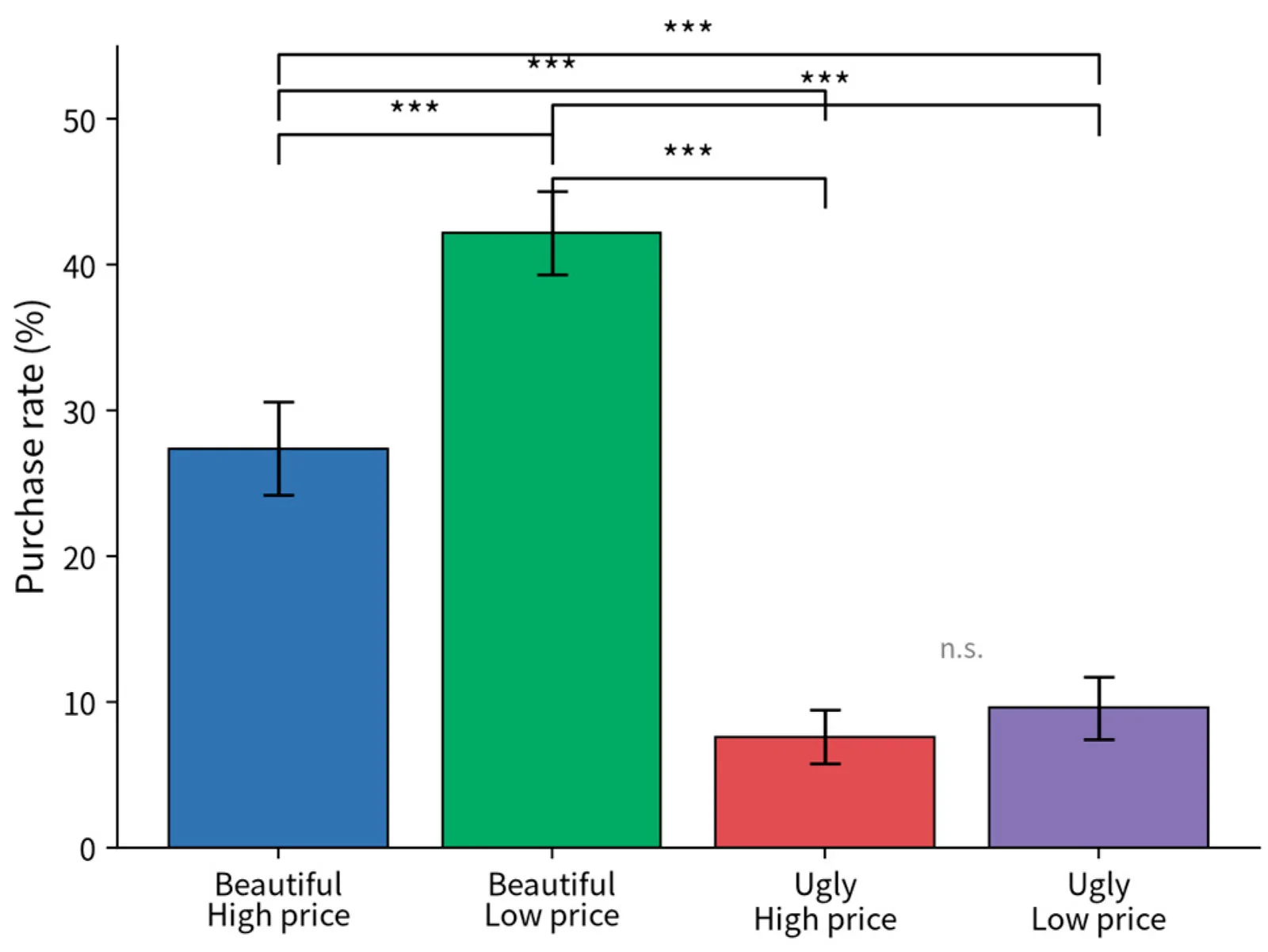
Mean purchase rates for the four conditions (BH, BL, UH, UL). Note. Error bars represent the standard error of the mean. Asterisks indicate significant pairwise differences after FDR correction. The n.s. indicates that the difference is not significant.

Mean purchase rates across the four conditions are reported in Table 3. Beautiful artworks were purchased more often than ugly artworks, and low prices increased purchase intention in every condition. A significant price by aesthetic interaction indicated that the low-price advantage was more pronounced for beautiful artworks. Among the six FDR-corrected pairwise comparisons, five were significant; the difference between ugly–high and ugly–low was not significant. As shown in Figure 2, aesthetic valence had a larger effect on purchase rates (*F*(1, 69) = 134.98, *p* < 0.001, *ηp*^2^ = 0.662) than price (*F*(1, 69) = 22.47, *p* < 0.001, *ηp*^2^ = 0.246).

Behavioural results supported H1, H2, and H3: low prices yielded higher purchase rates than high prices (H1), positively rated artworks yielded higher purchase rates than negatively rated ones (H2), and a significant price by aesthetic interaction was observed (H3).

### 4.2. ERP Results

**Figure 3:**
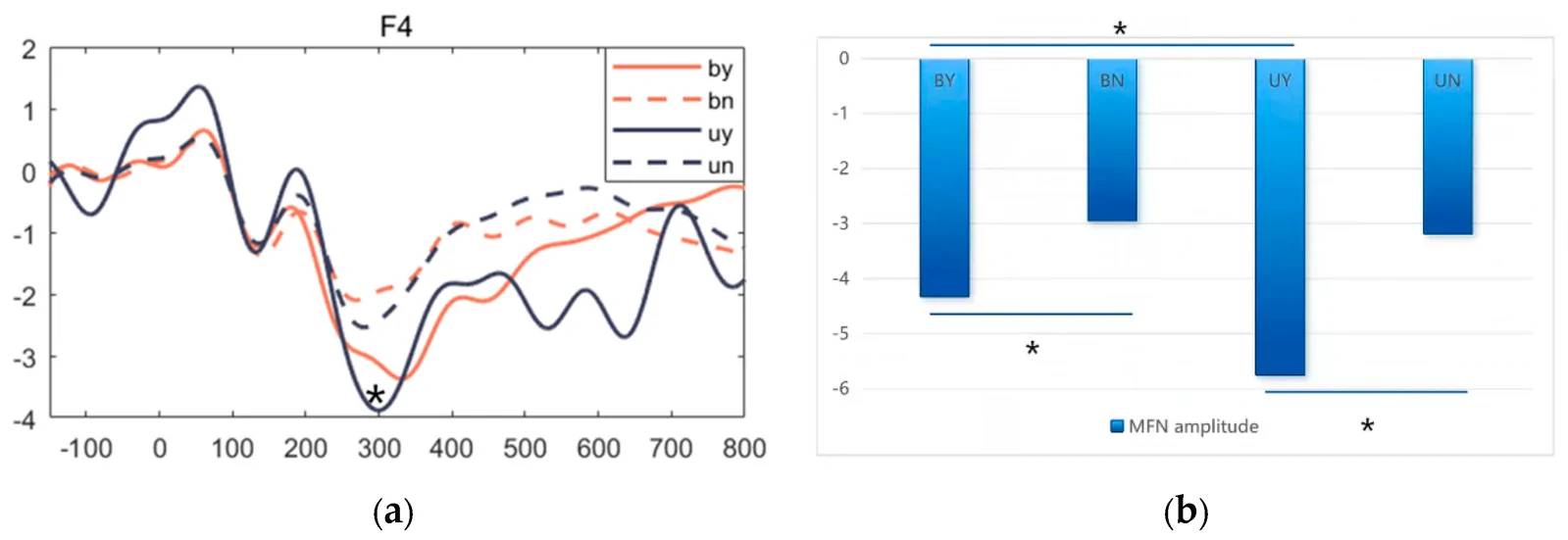
ERP results. Note. (**a**) ERP waveforms at F4 for the four conditions. (**b**) Mean MFN amplitudes (220–350 ms). Asterisks indicate significant differences after FDR correction. The BY analysis excluded 3 participants with no buy trials (N = 67); the UY analysis excluded 21 participants with no ugly-art buy trials (N = 49).

ERP waveforms were grouped into BY, BN, UY, and UN based on artwork type and decision. A 2 (aesthetic: beautiful vs. ugly) by 2 (decision: buy vs. reject) repeated-measures ANOVA on F4 MFN amplitude was conducted in the 49 participants with trials in all four cells (Figure 3). Both main effects and the interaction were significant (Table 4). Purchase trials elicited larger amplitudes than rejection trials, and ugly artworks elicited larger amplitudes than beautiful artworks, with the aesthetic difference more pronounced during purchase than rejection. Post hoc paired *t*-tests showed that the decision effect was significant for both beautiful and ugly artworks, whereas the aesthetic effect was significant only during purchase trials and not during rejection.

### 4.3. PSD Results

**Figure 4:**
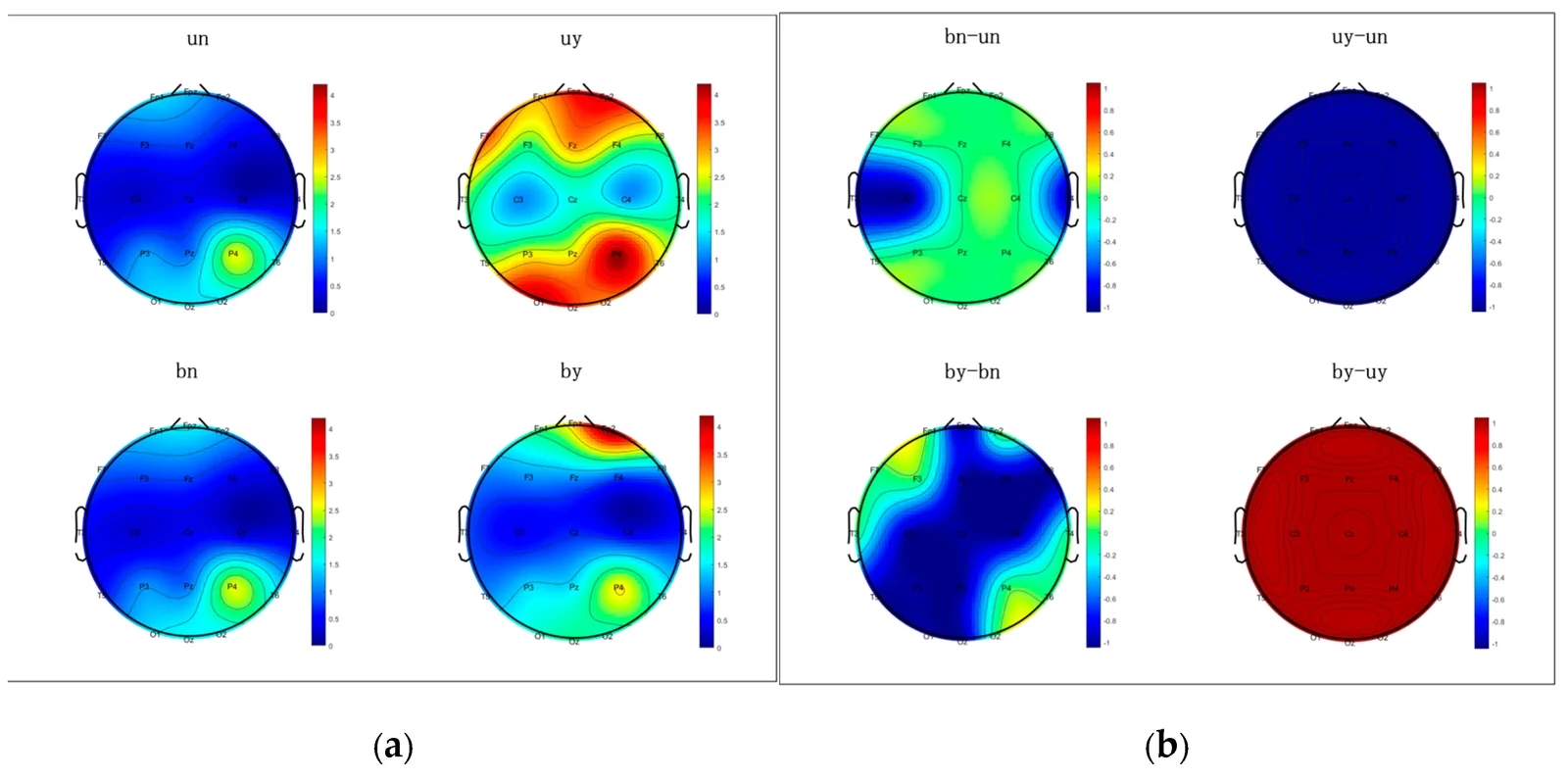
Theta-band (4–7 Hz) PSD analysis. Note. (**a**) Topographic maps of theta power for the four conditions. (**b**) Difference maps. Positive values indicate higher power in the former condition; negative values indicate higher power in the latter. Channel-wise, paired *t*-tests across 21 electrodes, FDR-corrected.

Topographic maps of theta power for the four conditions are shown in Figure 4. Overall, theta power was highest when participants bought ugly artworks. Right parietal theta power was relatively elevated across all four conditions. For beautiful artworks, purchase was accompanied by higher theta power than rejection in the central, left parietal, and right frontal regions. For ugly artworks, purchase was accompanied by higher theta power than rejection across the whole brain. Among purchase trials, ugly artworks elicited higher theta power than beautiful artworks across the whole brain. Among rejection trials, beautiful artworks elicited higher theta power only in the temporal lobe. Difference maps were based on channel-wise, paired *t*-tests across 21 electrodes, FDR-corrected, with the colour bar scaled in μV^2^/Hz.

A 2 (aesthetic) by 2 (decision) repeated-measures ANOVA on frontal theta power was conducted in the 49 participants with trials in all four cells. Both main effects and the interaction were significant (Table 5). Purchase trials showed higher theta power than rejection trials, ugly artworks showed higher theta power than beautiful artworks, and the aesthetic difference was larger during purchase trials.

### 4.4. Correlation Results

After testing the hypotheses above, we conducted exploratory correlation analyses examining how neural and behavioural measures varied with individual price-reference variables (Table 6). Three of the nine reached the 0.05 significance level: BH purchase rate was positively correlated with expected personal annual income in five years; BY MFN amplitude was negatively correlated with BH purchase rate; and monthly consumption expenditure was negatively correlated with theta power in both the UN and UY conditions. UY MFN amplitude was negatively correlated with annual household income, but this association did not reach significance (*r* = −0.269, *p* = 0.062). Because one participant did not report monthly consumption expenditure, the correlation between UY theta power and monthly consumption expenditure included only 48 participants.

Correlations between annual household income and purchase rates across the four conditions were small and non-significant. Because price levels were anchored to individual income, purchase rates would be expected to vary with income if income altered the experience of price. The small, non-significant correlations between purchase rates and income indicate that income did not substantially change participants’ response patterns to the high- and low-price categories, and the error arising from the income–price interaction was well controlled in this study. Household income was related to the conflict-related affect experienced when buying ugly artworks; in this case the income–price interaction was less well controlled, and participants with higher household income showed lower conflict-related affect when buying ugly artworks.

Based on these correlation analyses, we draw the following exploratory conclusions for future researchers to examine further. First, when facing a disliked but affordable artwork, brain activity may be more pronounced when monthly consumption expenditure is low. Theta power related to deciding whether to buy an ugly artwork was negatively correlated with monthly consumption expenditure, suggesting that individual differences in monthly consumption expenditure may relate to the degree of brain activity recruited when weighing whether to buy an ugly artwork. Second, spending on durable or symbolic goods may depend more on long-term expected income than on current cash on hand (Friedman, 1957). The task price was anchored to each participant’s current annual household income, whereas the variable correlated with BH purchase rate was expected personal annual income five years from now, a distinct construct. The positive association between BH purchase rate and expected future income may arise because students at this university generally experience income growth after graduation; therefore, although their current disposable income as students is limited, those with higher expected future incomes may still be willing to consider purchasing expensive artworks.

## 5. Discussion

### 5.1. Interpretation of Findings

When consumers decide whether to purchase an artwork, their brains process two considerations simultaneously: whether they like the piece (aesthetic attitude) and whether the price is worth paying (economic attitude). Consumer theory has examined how price affects purchasing, and neuroaesthetics has examined brain responses to beauty, but the two literatures have rarely intersected. The present study placed aesthetic and economic attitudes within a single purchase task while recording EEG, observing brain activity when the two attitudes were aligned and when they conflicted. Five hypotheses were derived and all five were supported.

At the behavioural level, beautiful artworks were purchased more often than ugly artworks, and low-priced artworks were purchased more often than high-priced ones. Aesthetic valence exerted a stronger effect than price (aesthetic main effect *F*(1, 69) = 134.98, *p* < 0.001, *ηp*^2^ = 0.662; price main effect *F*(1, 69) = 22.47, *p* < 0.001, *ηp*^2^ = 0.246). The interaction showed that the sales-promoting effect of low prices was larger for beautiful than for ugly artworks (interaction *F*(1, 69) = 25.87, *p* < 0.001, *ηp*^2^ = 0.273). For beautiful artworks, lowering the price from high to low raised purchase rates by about 15 percentage points; for ugly artworks, the corresponding increase was only about 2 percentage points. The most preferred condition was beautiful and low-priced art (*M* = 42.2%, *SD* = 24.2). Low prices reinforced an already positive aesthetic attitude but did little to reverse an already negative one; economic attitude acted on top of aesthetic attitude.

At the neural level, when participants made a purchase decision, both MFN amplitude and theta power were larger than when they made a rejection decision (MFN decision main effect *F*(1, 48) = 28.5, *p* < 0.001, *ηp*^2^ = 0.373; theta decision main effect *F*(1, 48) = 15.2, *p* < 0.001, *ηp*^2^ = 0.241). When participants chose to buy ugly artworks, both measures were larger than when they chose to buy beautiful artworks (MFN aesthetic main effect *F*(1, 48) = 22.1, *p* < 0.001, *ηp*^2^ = 0.315; theta aesthetic main effect *F*(1, 48) = 9.6, *p* = 0.003, *ηp*^2^ = 0.167; ugly buy vs. ugly reject *t*(48) = 4.31, *p* < 0.001, *d* = 0.61). The initial tendency is to reject, especially for ugly artworks. When the price is low enough that buying becomes justifiable, the brain may need to suppress the initial rejection tendency and execute the opposite response; the larger MFN amplitude observed on purchase trials is consistent with this interpretation. In the ERP literature, the MFN and feedback-related negativity have been associated with feedback processing and expectation violation, and frontal theta power has been linked to conflict monitoring and cognitive control. The higher theta power during purchase, especially when buying ugly artworks, is likewise consistent with greater conflict-resolution demands when aesthetic and economic attitudes diverge. Because the present design contrasts naturally occurring purchase and rejection decisions, these interpretations rest on associations and do not directly establish the underlying cognitive processes.

The exploratory correlations provided a preliminary characterisation of individual differences. BH purchase rate was positively correlated with expected personal annual income in five years (*r* = 0.263, *p* = 0.037); BY MFN amplitude was negatively correlated with BH purchase rate (*r* = −0.301, *p* = 0.014); UY MFN amplitude was negatively correlated with annual household income, although this association did not reach significance (*r* = −0.269, *p* = 0.062); and monthly consumption expenditure was negatively correlated with theta power in both the reject-ugly and buy-ugly conditions (*r* = −0.259, *p* = 0.031; *r* = −0.305, *p* = 0.036). These associations were exploratory and are presented as preliminary indications of how neural activity during purchase may vary across consumers with different income and spending levels.

Overall, purchasing art occurred most often when aesthetic and economic attitudes aligned. When the two pointed in the same direction, decisions required little neural effort; when they conflicted, the brain recruited cognitive control to resolve the conflict state. Prior neuroaesthetics research has recorded brain activity when people view art and find it pleasing, and prior consumer neuroscience research has recorded brain activity when people choose among products. The present study placed a painting and a price on opposite sides of the brain for comparison and measured the neural effort required to resolve the conflict between them.

### 5.2. Limitations

Several limitations should be noted. First, many stimuli were sourced from the National Art Museum of China and the China Art Exhibition Hall, and the sample consisted of young, non-art-trained students from a single western Chinese university. The concentration of the sample in terms of population and geography limits generalisability to established art markets, older art collectors, or different cultural contexts, as well as to broader consumer populations. Readers who wish to apply the conclusions to real consumption settings should verify them again. Future studies could recruit art-trained samples, expert samples, middle-aged or older samples, or samples from different cultural contexts. Second, relative price was anchored to participants’ self-reported annual household income, which was not independently verified. The high-price anchor of 86 times annual household income far exceeds the range of everyday art purchases; in real transactions, buyers of expensive art typically have correspondingly high incomes, so the price-to-income ratio would be lower. The low-price anchor of one-tenth of annual household income is also high for student participants whose actual disposable income is below their reported household income. These factors may introduce bias in the measurement of economic attitude. Third, the purchase task was hypothetical with no real payment, and images were presented for only 5 s, shorter than free viewing in a gallery. These factors may have depressed purchase rates relative to real buying; even under the most favourable condition, only 42.2% of trials resulted in a purchase, and the overall mean across the four conditions was 21%. Fourth, the aesthetic norming panel comprised only nine raters; although group-level ICC = 0.84 was acceptable, Fleiss’ kappa = 0.41 was moderate, and the panel was smaller than the commonly recommended 30 or more raters. A larger panel and longer presentation times might have yielded more robust beautiful/ugly categorisation. Fifth, the offline filter was limited to 0.1 to 30 Hz, which retained the theta and MFN bands but excluded very slow and very fast activity. Sixth, the MFN measurement differed from classic literature. Because the decision-related difference wave was searched across all 21 channels before hypothesis testing and peaked at the right frontal electrode F4, MFN amplitude was extracted there; classic MFN and FRN maxima are located at midline frontocentral sites, and the right frontal peak observed here is uncommon. The full 21-channel difference-wave topography was not archived, so readers cannot directly inspect the scalp distribution; future studies should retain channel-level amplitudes to permit midline reanalysis and region-of-interest averaging. Seventh, art training and art expertise may alter consumers’ decision processes, and all participants were non-art-major students, so the observed patterns likely reflect novices and may not generalise to professional art markets. Future studies should include art training questionnaires or expertise ratings. In addition, individual differences in aesthetic sensitivity, art appreciation, prior art experience, and art collection intention were not measured; whether the observed neural effects are moderated by aesthetic sensitivity remains an open question. Future studies could incorporate individual-difference measures to examine the influence of aesthetic sensitivity. Eighth, ANOVA power for the three contrasts exceeded 0.99, but the five exploratory correlations had power between 0.47 and 0.70. These neural associations should therefore be treated as suggestive rather than confirmatory.

## Figures and Tables

**Table 1:** Number of artifact-free trials per condition.

Condition	N	*M* (*SD*)	Minimum
BY (beautiful, buy)	67	20.9 (4.5)	12
BN (beautiful, reject)	70	39.1 (5.2)	28
UY (ugly, buy)	49	5.2 (2.8)	3
UN (ugly, reject)	70	54.8 (4.1)	42

**Table 2:** Sample sizes for each analysis.

Analysis	N
Behavioural ANOVA (purchase rates)	70
MFN ANOVA (participants with trials in all four cells)	49
Theta ANOVA (participants with trials in all four cells)	49
BY vs. BN (decision effect for beautiful)	67
UY vs. UN (decision effect for ugly)	49
UY vs. BY (aesthetic effect on purchase trials)	49
UN vs. BN (aesthetic effect on rejection trials)	70
Correlation analyses (N per coefficient; see the correlation analysis section.)	48–70

**Table 3:** Mean purchase rates and 2 × 2 repeated-measures ANOVA (N = 70).

Condition	M (%)	SD (%)
Beautiful–high (BH)	27.4	26.7
Beautiful–low (BL)	42.2	24.2
Ugly–high (UH)	7.6	15.4
Ugly–low (UL)	9.6	18.0
Effect	*F*(1, 69)	*ηp* ^2^
Price (high vs. low)	22.47 ***	0.246
Aesthetic (beautiful vs. ugly)	134.98 ***	0.662
Price × Aesthetic	25.87 ***	0.273

Note. *** indicates that the result is significant at a 1/1000 confidence level.

**Table 4:** Results from 2 × 2 repeated-measures ANOVA and post hoc pairwise comparisons for MFN amplitude at F4 (ANOVA N = 49; post hoc tests use available participants per group).

Effect/Contrast	Statistic	*p*	Effect Size
Decision main effect (buy vs. reject)	*F*(1, 48) = 28.5	<0.001	*ηp*^2^ = 0.373
Aesthetic main effect (ugly vs. beautiful)	*F*(1, 48) = 22.1	<0.001	*ηp*^2^ = 0.315
Decision × Aesthetic	*F*(1, 48) = 18.3	<0.001	*ηp*^2^ = 0.276
BY vs. BN (decision effect, beautiful)	*t*(66) = 5.12	<0.001	*d* = 0.63
UY vs. UN (decision effect, ugly)	*t*(48) = 4.31	<0.001	*d* = 0.61
UY vs. BY (aesthetic effect, buy)	*t*(48) = 4.73	<0.001	*d* = 0.68
UN vs. BN (aesthetic effect, reject)	*t*(69) = 1.42	0.160	*d* = 0.17

**Table 5:** Results from 2 × 2 repeated-measures ANOVA for frontal theta (4–7 Hz) power (N = 49).

Effect	*F*(1, 48)	*p*	*ηp* ^2^
Decision (buy vs. reject)	15.2	<0.001	0.241
Aesthetic (ugly vs. beautiful)	9.6	0.003	0.167
Decision × Aesthetic	8.1	0.007	0.144

**Table 6:** Exploratory Pearson correlations among neural and behavioural measures and individual price-reference variables.

Pair	r	N	*p*
Annual household income × BH purchase rate	0.12	70	0.31
Annual household income × BL purchase rate	−0.08	70	0.51
Annual household income × UH purchase rate	0.06	70	0.62
Annual household income × UL purchase rate	−0.14	70	0.24
BH purchase rate × expected income in 5 years	0.263	70	0.037
BY MFN amplitude × BH purchase rate	−0.301	67	0.014
UY MFN amplitude × annual household income	−0.269	49	0.062
UN theta power × monthly consumption expenditure	−0.259	70	0.031
UY theta power × monthly consumption expenditure	−0.305	48	0.036

## Data Availability

The data presented in this study are openly available in Harvard Dataverse at https://doi.org/10.7910/DVN/PSY7ON and Supplementary Materials.

## Supplementary Materials

The following supporting information can be downloaded at: https://www.mdpi.com/article/10.3390/bs16101819/s1.
